# Metallodrugs in the battle against non-small cell lung cancer: unlocking the potential for improved therapeutic outcomes

**DOI:** 10.3389/fphar.2023.1242488

**Published:** 2023-08-31

**Authors:** Xianzhi Xu, Feng Dai, Yiting Mao, Kai Zhang, Ying Qin, Jiwei Zheng

**Affiliations:** ^1^ School of Stomatology, Xuzhou Medical University, Xuzhou, China; ^2^ Department of Cardiology, Affiliated Hospital of Xuzhou Medical University, Xuzhou, China

**Keywords:** non-small cell lung cancer, metallodrugs, chemotherapy, drug resistance, therapeutic strategies

## Abstract

Non-small cell lung cancer (NSCLC) remains a leading cause of cancer mortality worldwide. Platinum-based chemotherapy is standard-of-care but has limitations including toxicity and resistance. Metal complexes of gold, ruthenium, and other metals have emerged as promising alternatives. This review provides a comprehensive analysis of metallodrugs for NSCLC. Bibliometric analysis reveals growing interest in elucidating mechanisms, developing targeted therapies, and synergistic combinations. Classification of metallodrugs highlights platinum, gold, and ruthenium compounds, as well as emerging metals. Diverse mechanisms include DNA damage, redox modulation, and immunomodulation. Preclinical studies demonstrate cytotoxicity and antitumor effects *in vitro* and *in vivo*, providing proof-of-concept. Clinical trials indicate platinums have utility but resistance remains problematic. Non-platinum metallodrugs exhibit favorable safety but modest single agent efficacy to date. Drug delivery approaches like nanoparticles show potential to enhance therapeutic index. Future directions include optimization of metal-based complexes, elucidation of resistance mechanisms, biomarker development, and combination therapies to fully realize the promise of metallodrugs for NSCLC.

## Introduction

Non-small cell lung cancer (NSCLC), the most prevalent subtype of lung cancer, accounts for approximately 85% of all cases and remains a leading cause of cancer-related mortality worldwide (Hoang et al., 2019; Sung et al., 2021). The elevated mortality rate of NSCLC stems from challenges such as late-stage diagnosis, limited treatment options, and the emergence of drug resistance (Hirsch et al., 2017). Thus, the urgent need for innovative therapeutic strategies is evident.

Current treatment modalities for NSCLC, including surgery, chemotherapy, radiotherapy, targeted therapy, and immunotherapy, provide a diverse approach to management (Herbst et al., 2018). Significant advances in targeted therapies and immunotherapies have resulted in several effective agents that target specific molecular pathways or modulate immune response against cancer cells (Hendriks et al., 2023). Nevertheless, chemotherapy remains a fundamental component of NSCLC treatment, especially for patients without actionable molecular alterations or those experiencing progression following targeted treatments. However, the effectiveness of chemotherapy can be compromised due to drug resistance, adverse effects, and a narrow therapeutic index, emphasizing the need for novel therapeutic agents (Zhou et al., 2023).

Metallodrugs have emerged as a promising class of anti-cancer agents, offering unique mechanisms of action and potential to overcome drug resistance (Mokesch et al., 2020; Goldberg et al., 2022; Huang et al., 2022). Famous examples such as cisplatin have been widely used in treating various cancers, including NSCLC, but their clinical utility is limited by toxicity profiles and the development of resistance mechanisms in cancer cells. Consequently, exploration of alternative metal-based compounds, such as gold, ruthenium, copper, and iron complexes, is underway, as they have demonstrated anti-cancer activity in preclinical investigations (Ude et al., 2019; Carneiro et al., 2020; Tunes et al., 2020; Lu N. et al., 2022). These non-platinum metallodrugs possess potential to circumvent limitations of platinum-based agents, exhibiting different modes of action, reduced toxicity, and ability to overcome resistance mechanisms (Kar et al., 2021; Komarnicka et al., 2021; Li F. et al., 2022; Xiao et al., 2022).

This review aims to provide a comprehensive analysis of the current landscape of metallodrugs in NSCLC treatment. We will commence with a bibliometric analysis of metallodrugs in NSCLC research from 2010 to 2023, followed by a critical evaluation of preclinical study methodologies, and a discussion of the controversies and inconsistencies in the research findings. We will highlight the most promising metallodrugs and identify the gaps in the current knowledge and the challenges that need addressing for future research. Through this consolidated review of recent research findings, we aspire to underscore the potential advantages and challenges associated with metallodrug therapy in NSCLC and stress the importance of ongoing research and collaboration to propel the advancement and clinical implementation of these innovative therapeutic agents.

## Methodology

### Search strategy

PubMed, Scopus, and Web of Science databases were used to conduct systematic searches of the literature. The search was restricted to articles published between 1 January 2010, and 1 March 2023, to ensure a focus on the latest developments in the field. The search terms used included “non-small cell lung cancer,” “non-small cell lung cancer,” “non-small cell lung carcinoma,” “non-small-cell lung carcinoma,” “carcinoma, non-small cell lung,” “NSCLC; ” “metallodrugs,” “metallo-drugs,” “metal-based drugs,” “cisplatin,” “carboplatin,” “oxaliplatin,” “auranofin,” “gold complexes,” “ruthenium complexes,” “NAMI-A,” “KP1019,” “KP1339,” and “nanotechnology.” Boolean operators (AND, OR) were employed to combine search terms, and appropriate filters were applied to refine the search results.

### Inclusion and exclusion criteria

Consideration was given to the inclusion of articles in this review if they met the following criteria: 1) original research articles, reviews, or clinical trial reports. 2) studies focused on the use of metallodrugs for the treatment of NSCLC; 3) articles published in English; and 4) articles with accessible full-text versions. Exclusion criteria were as follows: 1) articles not related to the treatment of NSCLC with metallodrugs; 2) articles published in languages other than English; 3) conference abstracts, editorials, commentaries, or letters without sufficient data; and 4) duplicate publications.

### Data extraction

Two independent reviewers screened the identified articles’ titles and abstracts to assess their inclusion eligibility. Any disagreement between the reviewers was settled by discussion and, if required, through consultation with a third reviewer. The full texts of eligible articles were then carefully reviewed, and the following information was extracted: 1) study design and objectives; 2) type of metallodrugs investigated; 3) mechanisms of action; 4) preclinical or clinical findings; 5) drug delivery systems or nanotechnology approaches; and 6) conclusions and future perspectives.

## Results

### Bibliometric analysis of metallodrugs in non-small cell lung cancer research from 2010 to 2023

**FIGURE 1:**
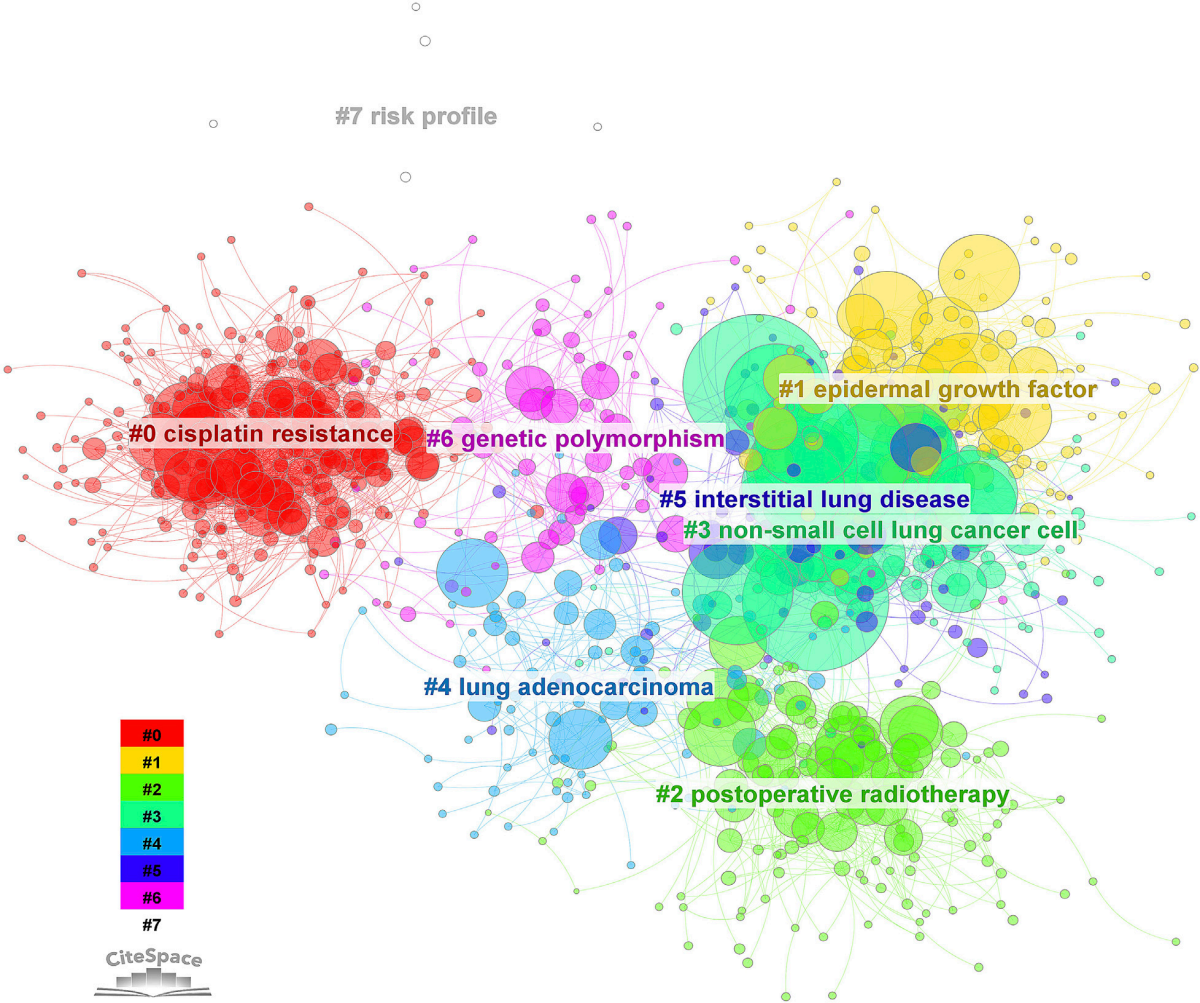
Cluster analysis of keyword citation related to metallodrugs and NSCLC from 2010 to 2023. A bibliometric analysis was performed using CiteSpace 6.2.R2 on 7276 articles related to metallodrugs combined with NSCLC. The node type was set to “keyword” and the clustering algorithm was based on co-occurrence frequency. The resulting clusters were labeled with ATK (abstract, title, keyword) and numbered as follows: 0. cisplatin resistance, 1. EGF, 2. postoperative radiotherapy, 3. NSCLC, 4. lung adenocarcinoma, 5. interstitial lung disease, 6. genetic polymorphism, 7. risk profile. The size of each node represents the frequency of the keyword, and the color indicates the corresponding cluster.

The bibliometric analysis (CiteSpace 6.2.R2 (64-bit) Advanced) underscores major research areas, which include overcoming cisplatin resistance, developing targeted therapies like EGFR inhibitors, and exploring the potential synergistic effects of combining metal drugs with other treatments such as radiotherapy, immunotherapy, and personalized medicine (Figure 1). It also reveals a growing interest in understanding the molecular characteristics of specific NSCLC subtypes, such as lung adenocarcinoma, and developing novel metal-containing complexes.

**FIGURE 2:**
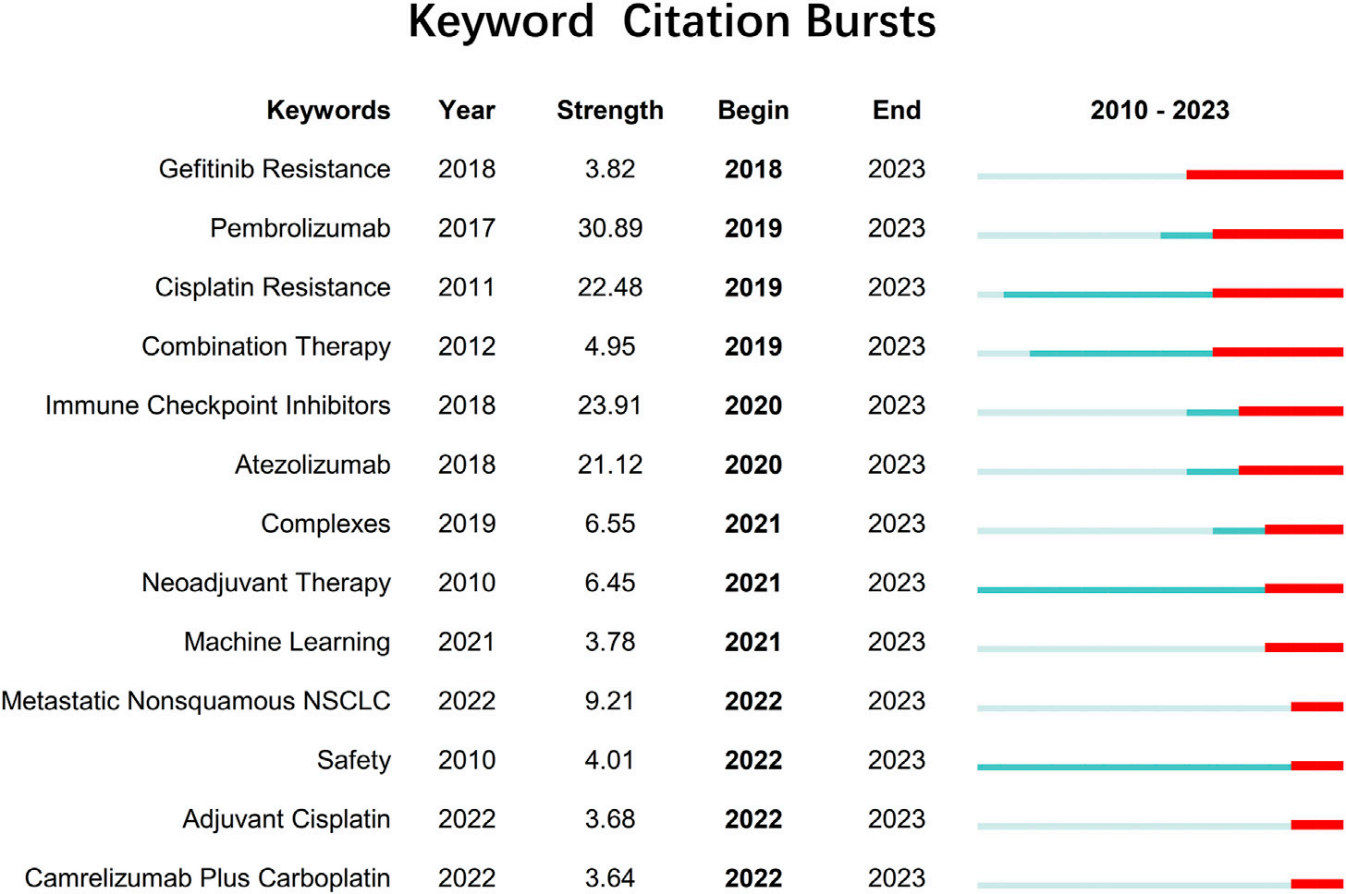
Keywords with citation bursts related to metallodrugs and NSCLC extending until 2023 from 2010 to 2023. Citation bursts are periods of time when a keyword is cited more frequently than expected based on its previous citation history. The table shows the keywords with citation bursts detected by CiteSpace 6.2.R2, along with the year of burst detection, the burst strength (a measure of citation intensity), and the burst duration (the start and end year of the burst).

With the integration of advanced computational techniques like machine learning and data-driven approaches, researchers are identifying new metal drug candidates and optimizing treatment strategies for NSCLC. The citation bursts (Figure 2) for metastatic nonsquamous NSCLC, safety adjuvant cisplatin, and camrelizumab plus carboplatin demonstrate a growing interest in understanding and developing effective treatments for specific subgroups of patients, evaluating the safety profile and potential toxicities of adjuvant therapies, and exploring the combination of immune checkpoint inhibitors with traditional chemotherapy agents.

Continued research in these areas will be essential for improving the outcomes of patients with NSCLC and overcoming challenges posed by drug resistance, molecular heterogeneity, and treatment-related complications. Emphasis on understanding the safety and efficacy of combination therapies, the impact of genetic polymorphisms on metal drug response, and the development of personalized treatment strategies will further enhance the therapeutic landscape for NSCLC patients.

### Classification of metallodrugs

**FIGURE 3:**
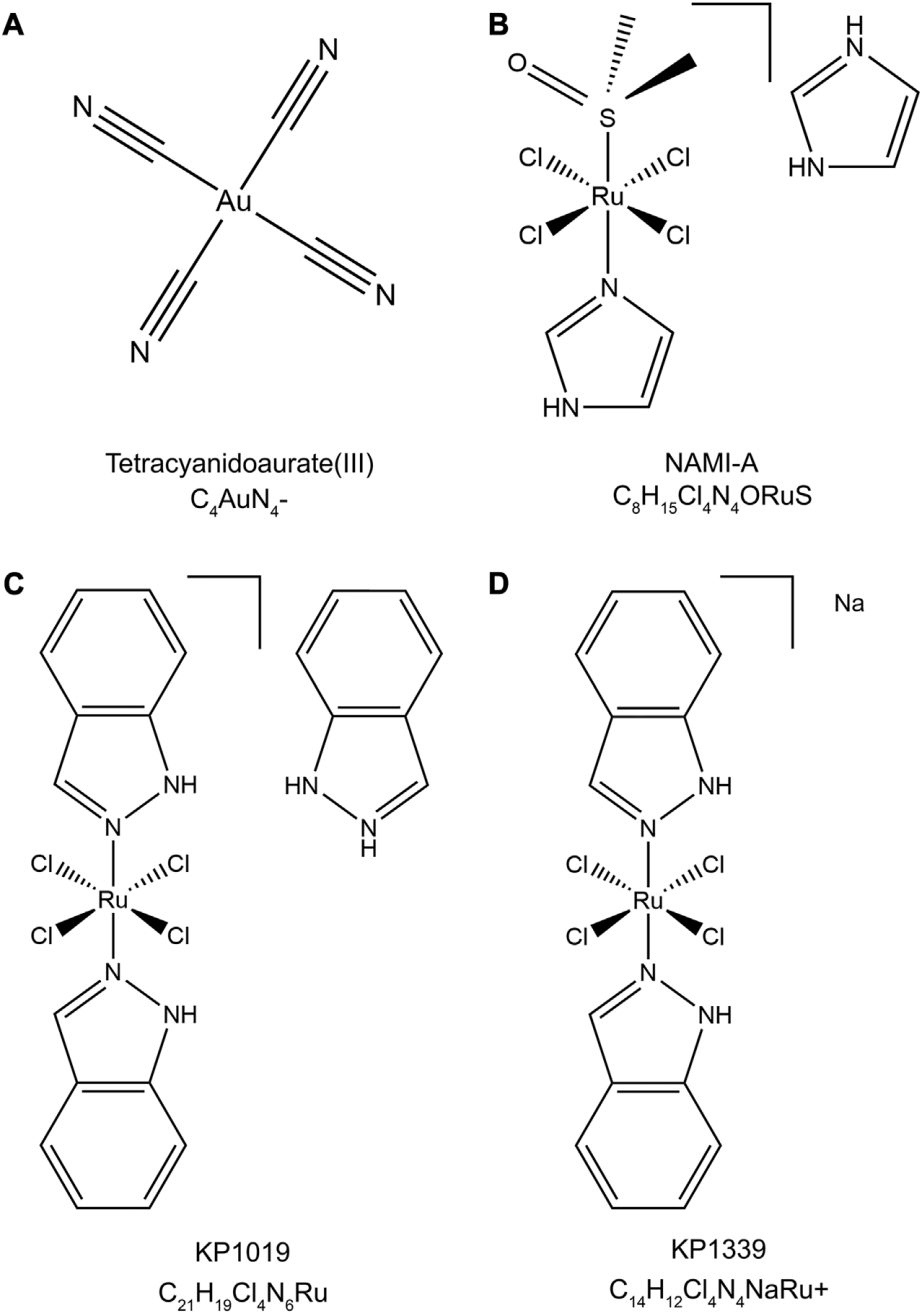
Chemical structures and molecular formulas of selected uncommon drugs. **(A)** Tetracyanidoaurate (III) (PubChem CID: 5460494), **(B)** NAMI-A (PubChem CID: 133082692), **(C)** KP1019 (ChEBI ID: 77,760), and **(D)** KP1339 (PubChem CID: 9806062). The 2D structures were retrieved from the PubChem or ChEBI databases and visualized using Chem3D (version 22.2). The style of the chemical structures was referenced from (Anthony et al., 2020).

**FIGURE 4:**
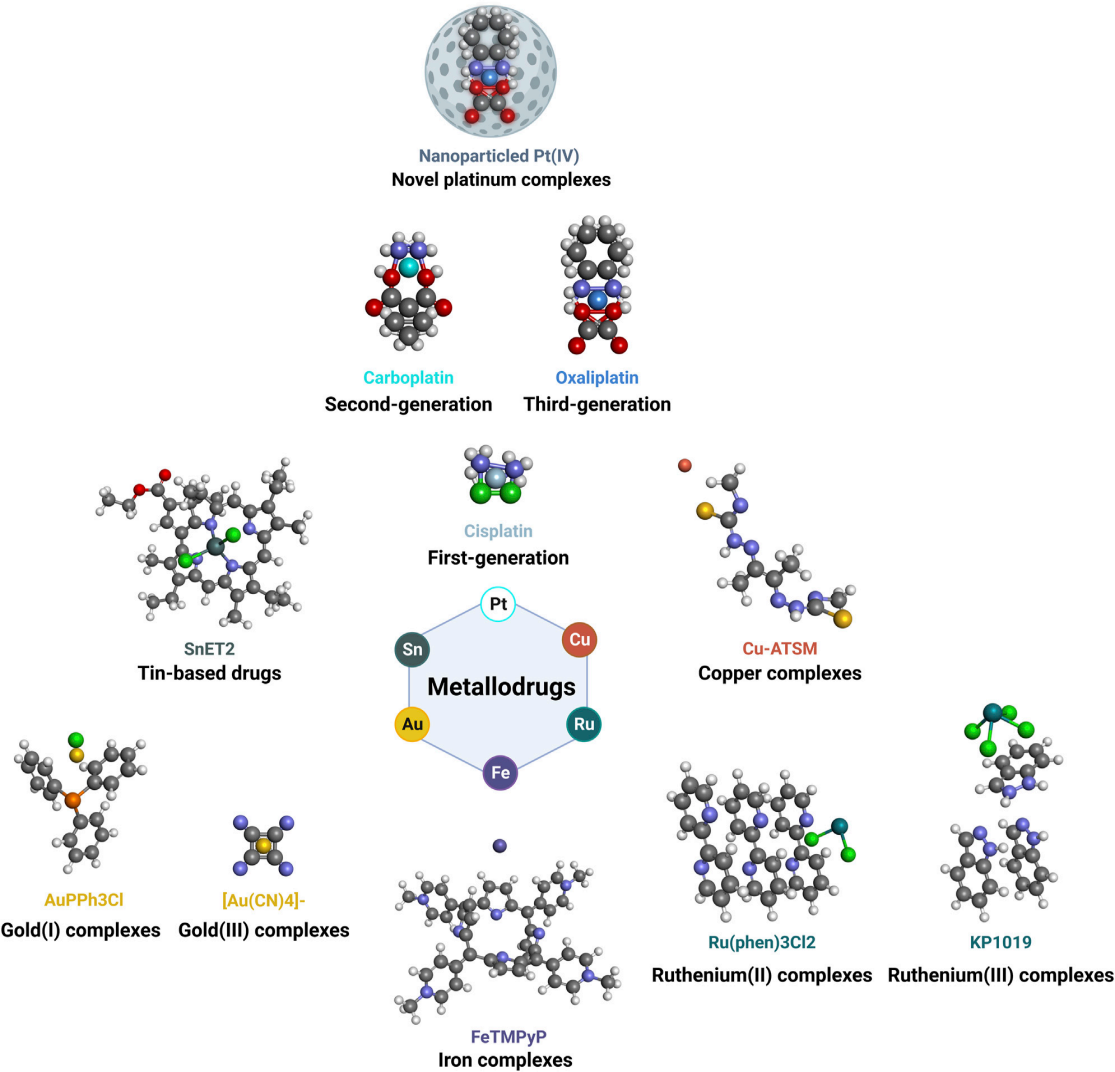
Schematic diagram of main classes of metallodrugs. The color of the metal atoms is roughly the same as the labeled color of the molecule name.

Metallodrugs are a diverse class of compounds containing metal ions with potential therapeutic applications in cancer treatment, including NSCLC. Their anti-cancer properties stem from their unique chemical and physical properties, which enable them to interact with various cellular targets and induce cytotoxic effects. We provide a comprehensive overview of the main classes of metallodrugs investigated for NSCLC treatment, including platinum, gold, ruthenium, and other metal-based compounds and their subcategories, potential advantages, and limitations. The structures and chemical formulas of certain uncommon drugs are shown in Figure 3, and the schematic diagram of the main classes of metallodrugs is shown in Figure 4. Besides, the molecular formula, metal core, reported advantages, reported limitations, and studies carried out for key metallodrugs for NSCLC treatment are summarized in Table 1.

**TABLE 1:** Key metallodrugs for NSCLC treatment.

Metallodrug	Molecular Formula	Metal Core	Reported Advantages	Reported Limitations	Studies Carried Out
Cisplatin	[Pt(NH_3_)_2_Cl_2_]	Platinum	Induces DNA damage and apoptosis; standard of care for NSCLC	High toxicity, resistance, poor selectivity	Clinical trials and preclinical studies
Oxaliplatin	[Pt(DACH)(oxalate)]	Platinum	Induces DNA damage and apoptosis; synergizes with anti-PD-1/PD-L1 antibodies; less nephrotoxic than cisplatin	Neurotoxicity, resistance, poor selectivity	Clinical trials and preclinical studies
Auranofin	[Au(PEt_3_)(SC_2_H_4_COOH)]	Gold(I)	Inhibits thioredoxin reductase; induces mitochondrial dysfunction and oxidative stress; overcomes cisplatin resistance; synergizes with PD-L1 blockade	Low bioavailability, high protein binding, off-target effects	Preclinical studies
[Au(bipydmb-H)Cl_2_]	[Au(bipydmb-H)Cl_2_]	Gold(III)	Causes DNA damage and inhibits topoisomerase II; induces apoptosis; higher cytotoxic potency than cisplatin in NSCLC cells	Stability issues, high toxicity, resistance mechanisms	Preclinical studies
NAMI-A	[Ru(η6-p-cymene)Cl_2_(ImH)]	Ruthenium(II)	Inhibits metastasis and angiogenesis; low toxicity and high selectivity; active against cisplatin-resistant cells	Low cytotoxicity, slow action, limited bioavailability	Clinical trials and preclinical studies
KP1019	[Ru(η6-p-indazole)Cl_2_(IndH)]	Ruthenium(III)	Induces cell cycle arrest and apoptosis; activates caspases and PARP cleavage; similar or lower IC50 than cisplatin in NSCLC cells	High toxicity, poor solubility, resistance mechanisms	Clinical trials and Preclinical studies
[SnCl_2_(HL1)]	[SnCl_2_(HL1)] (HL1 =aroylhydrazone ligand)	Tin(IV)	Inhibits DNA synthesis and induces apoptosis; increases ROS levels and reduces mitochondrial membrane potential; lower IC50 than cisplatin in NSCLC cells	Low stability, high toxicity, limited studies	Preclinical studies
Casiopeínas III-ia and III-Ea	[Cu(N-N)(O-O)] (N-N =tetradentate ligand; O-O =bidentate ligand)	Copper(II)	Induce apoptosis by increasing ROS levels and free calcium; reduce mitochondrial membrane potential; activate caspases; cause cell cycle arrest	High toxicity, low selectivity, stability issues	Preclinical studies
Deferoxamine	C_25_H_48_N_6_O_8_ (iron chelator)	Iron(III)	Inhibits tumor growth and angiogenesis by reducing iron availability; enhances the efficacy of radiotherapy and chemotherapy	Low bioavailability, high dose requirement, side effects	Clinical trials and preclinical studies
Cobalt protoporphyrin IX	C_34_H_32_CoN_4_O_4_ (hemeanalog)	Cobalt(II)	Induces heme oxygenase-1 expression; inhibits tumor growth and metastasis by modulating inflammation and immune response	Low solubility, high toxicity, limited studies	Preclinical studies
Palladium mesoporphyrin IX	C_34_H_32_PdN_4_O_4_ (hemeanalog)	Palladium(II)	Induces heme oxygenase-1 expression; inhibits tumor growth and angiogenesis by modulating inflammation and oxidative stress	Low solubility, high toxicity, limited studies	Preclinical studies
[Rh_2_(OAc)_4_]	[Rh_2_(OAc)_4_] (dirhodium tetraacetate)	Rhodium(II)	Inhibits tumor growth and metastasis by inducing ferroptosis; synergizes with cisplatin and erlotinib; active against cisplatin-resistant cells	High toxicity, poor solubility, stability issues	Preclinical studies

Note. NSCLC refers to non-small cell lung cancer. The table summarizes key metallodrugs used in NSCLC treatment, detailing their molecular formula, metal core, reported advantages, reported limitations, and studies carried out. Clinical trials include both completed and ongoing research studies. Preclinical studies refer to laboratory experiments conducted *in vitro* or in animal models. Reported limitations may include challenges related to toxicity, resistance, solubility, and other pharmacological aspects.

### Common metallodrugs

Platinum, gold, and ruthenium-based drugs have been extensively studied due to their unique properties and potential in cancer treatment. Each of these classes of drugs, while exhibiting distinct characteristics, also share common features, including the ability to interact with cellular targets in unique ways, offering potential advantages over conventional treatments.

#### Platinum-based drugs

These are the most extensively studied and clinically utilized metallodrugs in cancer therapy. This group includes first-generation cisplatin (Johnstone et al., 2016), second-generation carboplatin (Devanabanda and Kasi, 2023a), third-generation oxaliplatin (Devanabanda and Kasi, 2023b), and novel platinum nanoparticled complexes (Fu et al., 2023). By forming DNA adducts induced apoptosis, these platinum-based drugs exert their anti-cancer effects with varying pharmacokinetics, toxicity profiles, resistance mechanisms, and therapeutic indications. Novel platinum complexes are currently under investigation to enhance the efficacy and selectivity of platinum-based chemotherapy.

#### Gold-based drugs

Gold complexes have garnered interest due to their distinct chemical properties and mechanisms of action compared to platinum drugs (Goetzfried et al., 2020). This category includes Gold(I) complexes like Auranofin and Gold (III) complexes like tetracyanidoaurate (III) (Figure 3A) (Freire Boullosa et al., 2021; Kim et al., 2021). Recent advances in gold-based drug design have led to new gold complexes with improved stability, selectivity, and efficacy.

#### Ruthenium-based drugs

Ruthenium complexes have emerged as a promising alternative to platinum-based drugs, owing to their unique coordination chemistry, lower toxicity, and distinct mechanisms of action (Glans et al., 2012). This group includes Ruthenium (II) and Ruthenium (III) complexes, each with different oxidation states, coordination chemistry, and action mechanisms. Ruthenium (II) complexes, such as those containing arene groups, have demonstrated selectivity towards cancer cells, effectively inducing apoptosis (Meng et al., 2019). Ruthenium (III) complexes, notably NAMI-A (Figure 3B) and KP1019 (Figure 3C), have shown potential in clinical trials for solid tumors, particularly in overcoming drug resistance (Dwyer et al., 2018).

NAMI-A, or Imidazolium-trans-tetrachloro (dimethylsulfoxide)imidazoleruthenium (III), features a central Ruthenium (III) atom coordinated by four chloride ions, one dimethyl sulfoxide (DMSO) molecule, and two imidazole rings in a trans configuration. This structure contributes to its anticancer activity, primarily inhibiting metastasis and angiogenesis in lung metastasis of solid tumors. KP1019, also known as Indazolium trans-[tetrachlorobis (1H-indazole) ruthenate (III)], is characterized by a central Ruthenium (III) atom coordinated by four chloride ions and two indazole rings in a trans configuration. KP1019 has shown promise in inducing apoptosis in cancer cells, even where traditional platinum-based drugs fail.

The development of novel ruthenium-based drugs continues, aiming to enhance the efficacy and specificity of ruthenium-based chemotherapy, heralding a new era of more effective and targeted cancer treatments.

### Emerging metal-based drugs

Beyond the commonly studied platinum, gold, and ruthenium, a variety of other metal ions have been incorporated into anti-cancer drug design. These metals, each possessing unique chemical properties and biological activities, present new avenues for exploration in the treatment of NSCLC.

#### Tin-based drugs

Tin (IV) complexes have been investigated for their potential cytotoxic effects against NSCLC cell lines (Pantelić et al., 2020). Preliminary studies have shown promising results *in vitro*. Potential mechanisms of action for these complexes include DNA binding, modulation of redox reactions, and interference with cellular signaling pathways (Liu et al., 2019). Further exploration of these complexes could open new pathways for the development of effective NSCLC treatments.

#### Copper-based drugs

Copper complexes have emerged as potential anti-cancer agents, with their mechanisms of action involving DNA damage, modulation of redox reactions, and inhibition of angiogenesis. Copper-based drugs, such as Casiopeina III-ia, have demonstrated promising results in preclinical studies against various cancer types, including NSCLC (Figueroa-DePaz et al., 2022). The unique properties of copper complexes, including their redox activity and ability to disrupt angiogenesis, make them attractive candidates for further research in the context of NSCLC.

#### Iron-based drugs

Iron complexes have been explored for their ability to selectively target cancer cells through redox-based mechanisms and modulation of iron metabolism. Some iron chelators, like DFO and Dp44mT, have demonstrated anti-cancer activity *in vitro* and *in vivo* by modulating intracellular iron levels and inducing oxidative stress (Asperti et al., 2023). Given the critical role of iron in cell proliferation and survival, these iron-based drugs could provide a novel approach to the treatment of NSCLC.

#### Other metal-ion-based drugs

Additional metal ions, such as cobalt, palladium, and rhodium, have been investigated for their potential anti-cancer properties (Frei et al., 2020). These metal complexes have demonstrated diverse mechanisms of action, including DNA binding, proteasome inhibition, and modulation of cellular signaling pathways, underscoring the potential of metal-based drugs as alternative or complementary treatment options for NSCLC (Serio et al., 2022).

Each of these metal ions offers unique chemical properties and potential biological activities that, with further research and development, could lead to innovative therapeutic strategies for overcoming the current challenges in NSCLC treatment, such as drug resistance and toxicity.

### Mechanisms of action

**FIGURE 5:**
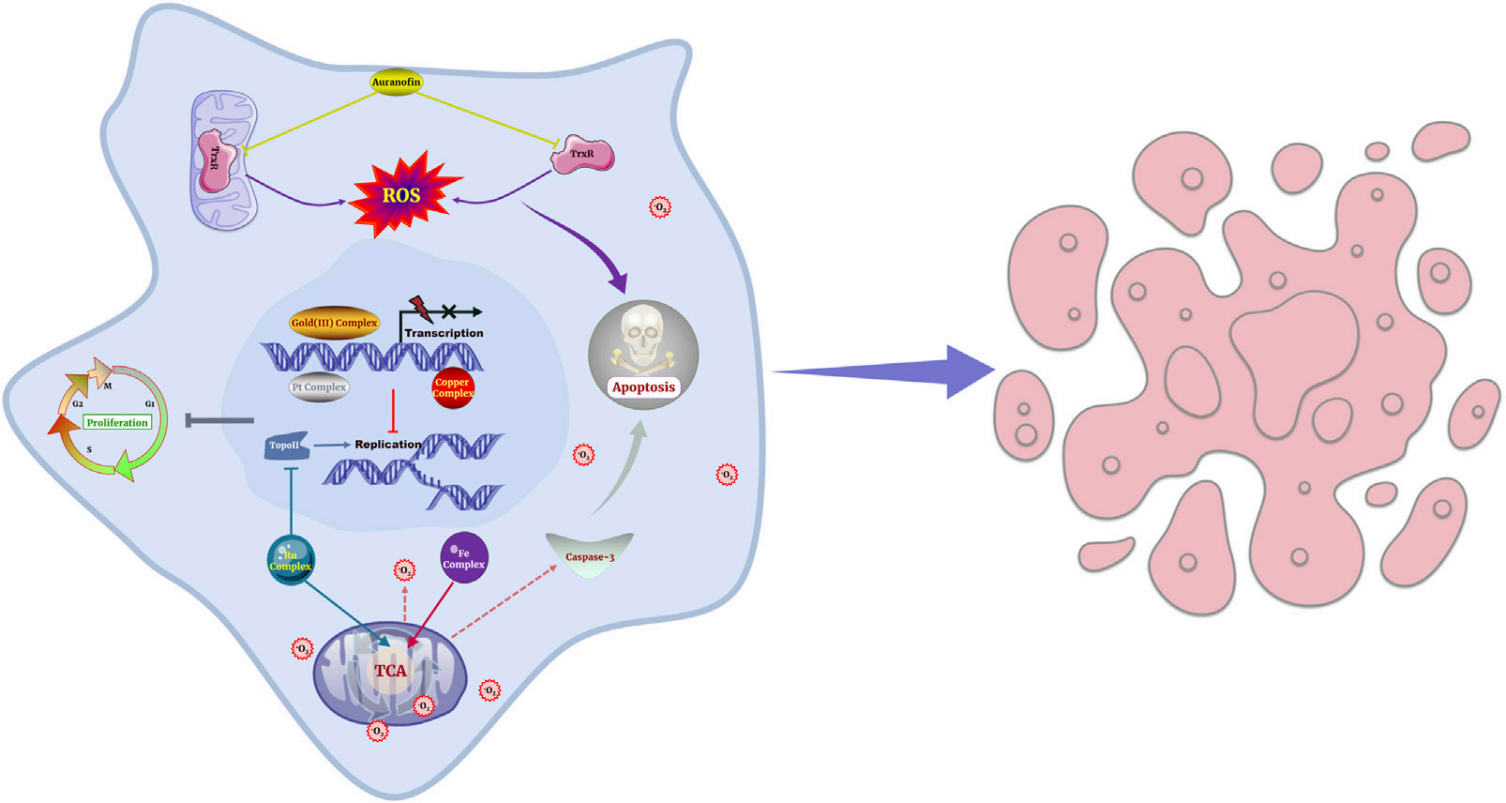
Schematic representation of the mechanisms of action of metallodrugs in NSCLC cells. These mechanisms are depicted in the context of NSCLC treatment, such as causing DNA damage and hindering DNA replication and transcription, inhibiting essential enzymes, modulating cellular redox processes and generating ROS.

Metallodrugs exhibit diverse anti-cancer mechanisms, but their efficacy is often limited by toxicity, resistance, and inconsistent activity across cancer types. Key mechanisms include DNA damage, redox modulation, angiogenesis inhibition, and immunomodulation, but optimal exploitation of these mechanisms requires deeper analysis. Figure 5 provides a visual representation of the mechanisms of action of metallodrugs in NSCLC cells.

DNA-damaging platinum drugs like cisplatin are effective first-line therapies, but resistance frequently develops due to enhanced DNA repair, limiting their long-term efficacy (Golan Berman et al., 2021). Ruthenium complexes like [Ru (η5-C5H4CH2OH)(PPh3) (4,4′-di (hydroxymethyl)-2,2′-bipyridine)] [CF3SO3] damage DNA through minor groove binding rather than major groove lesions formed by cisplatin, suggesting potential to overcome platinum resistance (Teixeira-Guedes et al., 2022). However, clinical studies show their single-agent efficacy is modest at best (Dwyer et al., 2018). This highlights the need for combination strategies and predictive biomarkers to enhance activity.

Protein-targeting metallodrugs like the gold (I) thiol-reactive complex auranofin, an inhibitor of the selenoenzyme thioredoxin reductase (TrxR), have shown cytotoxic activity by disrupting redox homeostasis in preclinical studies (Merlino et al., 2017; Delgobo et al., 2021). But clinical activity as single agents is again limited, likely due to compensatory mechanisms that mitigate redox effects (Gandhi et al., 2011). Such agents may have greater success when combined with other stressors. For example, auranofin enhanced cisplatin activity in lung cancer cells by further potentiating oxidative stress (Cui et al., 2022; Johnson et al., 2023).

While metallodrugs can modulate diverse processes like cell cycle arrest and apoptosis (Zhao et al., 2019; Herrera-Ramírez et al., 2023), single-target effects may be insufficient for robust anticancer activity. Agents affecting multiple pathways, like multi-target ruthenium complexes, may be more effective (Li Y. L. et al., 2022). Angiogenesis inhibitors also show promise, with ruthenium and gold complexes demonstrating anti-angiogenic and anti-metastatic effects in preclinical models (Kwong et al., 2016; Elie et al., 2018). But resistance mechanisms involving alternative pro-angiogenic pathways may limit their efficacy.

Immunomodulation is an emerging hallmark of metallodrugs, with platinum agents and gold complexes modulating immune cell function and expression of immune checkpoint proteins (Lesterhuis et al., 2011; Ray and Mukherjee, 2022). This sensitizes tumors to immune-mediated killing. Combining metallodrugs with immunotherapies like checkpoint inhibitors may provide synergistic benefits.

Overall, current metallodrugs demonstrate promising anti-cancer capabilities through diverse mechanisms, but single-agent efficacy is constrained by compensatory pathways that limit durable responses. A multi-targeted approach combining cytotoxic, angiogenic, and immunomodulatory agents appears most likely to overcome these limitations and achieve more potent anti-tumor activity.

### Preclinical studies

Preclinical studies provide critical insights into the potential efficacy, selectivity, and mechanisms of action of metallodrugs for NSCLC treatment. These studies encompass both *in vitro* and *in vivo* investigations using cancer cell lines and animal models. However, it is important to note that the results of these studies should be interpreted with caution due to potential limitations and biases inherent in preclinical research.

### *In Vitro* studies

#### Cytotoxicity

Numerous *in vitro* studies have reported the cytotoxic effects of different metallodrugs against NSCLC cell lines. Platinum drugs like cisplatin, carboplatin, and oxaliplatin have exhibited potent cytotoxicity, attributed to their ability to form DNA adducts and trigger apoptosis (Zhang et al., 2022). However, platinum agents have known limitations such as toxicity, resistance, and poor selectivity. Consequently, there is interest in evaluating alternative metallodrugs. For instance, the gold(I) complex auranofin displayed cytotoxicity and overcame cisplatin resistance in H1993 NSCLC cells by inducing mitochondrial dysfunction and oxidative stress (Cui et al., 2022). Besides, specific gold (III) derivatives have exhibited greater cytotoxic potency against NSCLC cells compared to cisplatin. For example, the gold (III) complex [Au (bipydmb-H)Cl2] demonstrated 2-fold greater cytotoxicity than cisplatin in A549 NSCLC cells in one study (Carboni et al., 2018). While promising, small sample sizes, lack of comparison groups, and limited generalizability temper the strength of conclusions from these early studies. More rigorous evaluations of cytotoxic potential and advantages over existing agents are still needed.

Ruthenium complexes like NAMI-A and KP1019 also reduced NSCLC cell viability, but direct comparisons to platinum drugs have been limited (Bergamo and Sava, 2011). Interestingly, specific copper complexes such as Casiopeínas III-ia and III-Ea, and organotin (IV) complexes containing aroylhydrazone ligands exhibited cytotoxicity too, but these studies had very small sample sizes (n < 10) and lacked proper dose-response assessments; The organotin (IV) complexes demonstrated inhibition of DNA synthesis and induction of apoptosis in H460 NSCLC cells (Jo and Onwudiwe, 2018; Silva-Platas et al., 2018; Pantelić et al., 2020; Figueroa-DePaz et al., 2022). Overall, metallodrugs display cytotoxic promise *in vitro*, but larger scale studies with improved methodological rigor are required to elucidate their therapeutic potential over current standards of care.

#### Selectivity

Selectivity towards cancerous over normal cells is vital to limit chemotherapy side effects. Certain ruthenium and copper complexes have shown selectivity in small *in vitro* studies, attributed to unique targets and mechanisms compared to platinum drugs. For example, a ruthenium (II) complex with 2,2′-bipyridine and 2-pyridinecarboxylic acid ligands (RuBPCA) exhibited cytotoxicity against human breast cancer cells (MCF-7) and human cervical cancer cells (HeLa) by inducing apoptosis and cell cycle arrest (Khater et al., 2022). The complex also showed higher selectivity index than cisplatin, suggesting lower toxicity to normal cells (Khater et al., 2022). Similarly, a copper (II) complex with 2,9-dimethyl-1,10-phenanthroline and 2-hydroxybenzoic acid ligands (CuDMPhenSA) displayed anticancer activity against human colon cancer cells (HCT116) and human lung cancer cells (A549) by generating reactive oxygen species and disrupting mitochondrial membrane potential (Gu et al., 2022). The complex also exhibited higher selectivity index than cisplatin and lower resistance factor than oxaliplatin, indicating better efficacy and lower resistance (Gu et al., 2022). However, selective cytotoxicity has not been extensively investigated, with most studies focusing on efficacy rather than selectivity. Moreover, the specificity of effects has been examined in very few cell lines, and normal cell controls are often lacking. Further *in vitro* research with larger sample sizes, proper normal cell comparators, and more metallodrug classes is essential to substantiate and extend early selectivity findings.

#### Cellular uptake, subcellular localization, and biomolecular targets

Understanding metallodrug transport, localization, and interactions is key to elucidating their anticancer mechanisms. As mentioned in the previous section, Platinum agents like cisplatin accumulate in the nucleus and form DNA adducts, and Gold (III) complexes localize in the nucleus and mitochondria, targeting DNA and redox proteins respectively. Besides, Ruthenium complexes exhibit diverse distribution patterns depending on structure, interacting with DNA, proteins, and enzymes (Elgar et al., 2023). While illustrative, most localization studies use only one or 2 cell lines. Testing in a broader range of NSCLC models would provide more robust, generalizable insights into subcellular trafficking. High-throughput approaches could also help better characterize metallodrug targets across the proteome and genome (Galvez et al., 2018). Overall, preliminary uptake and target studies show promise but require expanded validation.

#### Molecular and cellular mechanisms

*In vitro* investigations have unraveled metallodrug effects on pathways related to DNA damage, redox signaling, cell cycle, apoptosis, angiogenesis, and immune modulation (Tchounwou et al., 2021). Still, mechanistic studies are often limited to one or two signaling molecules or processes. Holistic profiling of genomic, proteomic, and metabolomic changes would provide greater systems-level understanding of metallodrug mechanisms. Emerging tools like CRISPR screens, single cell sequencing, and bioinformatics modeling could help achieve such network-wide views (Wangpaichitr et al., 2021). Additionally, *in vitro* models cannot fully recapitulate the complexity of human tumors. More physiologically relevant 3D cultures, organoids, and patient-derived xenografts could improve translation of findings. While vital, current mechanistic studies have narrow scopes and employ reductionist models. Broader, systems-based approaches in more representative models would strengthen molecular understanding and clinical relevance.

### *In Vivo* studies and animal models

#### Efficacy

Animal models enable crucial *in vivo* evaluation of metallodrug efficacy. Compounds like NAMI-A and copper complexes reduced tumor growth and prolonged survival in murine NSCLC xenografts (Gu et al., 2016; Lu Y. et al., 2022). Some ruthenium complexes also exhibited greater activity than cisplatin in zebrafish (Karas et al., 2021). These studies provide encouraging proof-of-concept for metallodrugs. However, small sample sizes, lack of randomization, and limited endpoints temper the strength of conclusions. Rigorous studies with improved statistical designs, adequately powered cohort sizes, diverse xenograft models, and multiple efficacy measures are imperative to substantiate early findings and identify promising clinical candidates.

#### Pharmacokinetics, biodistribution, and toxicity

Assessing pharmacological and safety profiles *in vivo* will facilitate clinical translation. Initial rodent studies indicate favorable pharmacokinetics, biodistribution, and reduced systemic toxicity for some ruthenium and copper agents over platinum analogues (Pierre et al., 2018; Levina et al., 2022). These results are promising, but standardization of metal quantification assays, biomarker development, and testing across different administration routes is needed. Moreover, higher species like pigs and non-human primates may better predict human pharmacology and toxicology. While supporting further development, available pharmacology and toxicology data still have important limitations.

#### Combination therapies

Evaluation of synergistic effects with other modalities is crucial, as combination strategies often enhance outcomes in NSCLC. The cisplatin and erlotinib pairing exhibited increased efficacy in xenograft models *versus* either agent alone (Lee and Wu, 2015). Certain metallodrugs also demonstrated synergy with chemotherapy, immunotherapy, or targeted therapies in preliminary studies (Ke et al., 2017). However, thorough investigations of multiple combination and dosing regimens are lacking. Elucidating mechanisms of synergy could also optimize pairing and sequencing. *In vivo* studies support combined approaches, but more expansive evaluations are required to identify promising regimens for clinical evaluation.

### Clinical studies and trials

Translation of metallodrugs to approved NSCLC therapies remains a challenge despite emerging clinical investigations. While platinum agents are standard-of-care, non-platinum metallodrugs have undergone limited trials with modest efficacy to date. This section synthesizes the latest clinical findings and ongoing efforts to improve outcomes.

### Completed trials

Cisplatin and carboplatin are well-established chemotherapeutics for advanced NSCLC. However, cumulative toxicities like neuropathy and nephrotoxicity, limited duration of response, and universal acquired resistance constrain their utility. Attempts to develop improved metallodrugs have shown limited gains.

Early trials found ruthenium agents NAMI-A and KP1019 well-tolerated but with minimal single agent activity in solid tumors including NSCLC (Leijen et al., 2015). A phase I trial combining the gold (I) drug auranofin with pemetrexed for NSCLC demonstrated acceptable safety but modest clinical benefit (Kawano et al., 2018). While valuable first steps, such studies lacked power for definitive conclusions. Recent findings from a phase I trial found the gold (III) pyridine complex PR-104 exhibited favorable pharmacokinetics and potential efficacy in refractory solid tumors (Jameson et al., 2010). However, clinical advantage over standard platinums remains unproven.

### Ongoing trials

Numerous platinum combinations continue under study for NSCLC. However, trials of non-platinum metallodrugs are limited. A phase II trial is investigating the ruthenium (III) agent KP1339 (Figure 3D) plus gemcitabine for advanced NSCLC (NCT02591051), while a phase I study is evaluating KP1339 with paclitaxel (NCT04168721) (ClinicalTrials, 2023a).

Meanwhile, a phase I trial is assessing the gold (III) complex RK-ES001A as a radiation sensitizer for NSCLC (NCT05471484) (ClinicalTrials, 2023b). Such combinations may enhance efficacy, but require rigorous evaluation *versus* standard regimens. Defining optimal dosing and patient selection will be critical.

### Drug delivery systems and nanotechnology

The application of drug delivery systems and nanotechnology offers promising strategies for improving the therapeutic potential of metallodrugs in NSCLC treatment. These approaches can enhance drug solubility, stability, bioavailability, and tumor targeting while reducing systemic toxicity. Advanced drug delivery systems have the potential to revolutionize the way we administer metallodrugs, improving their overall efficacy and minimizing side effects.

Nanoparticle-based delivery systems have emerged as a promising approach to improve the pharmacokinetic properties and therapeutic index of metallodrugs. For example, cisplatin-loaded nanoparticles engineered with iron metal ions have demonstrated improved anti-tumor efficacy and reduced side effects compared to free cisplatin in preclinical studies (Mao et al., 2022). Similarly, ruthenium complexes encapsulated in liposomes or polymeric nanoparticles have shown enhanced tumor targeting and anti-cancer activity *in vitro* and *in vivo* (Shen et al., 2017; Ma et al., 2019). The development of such nanoparticle-based delivery systems has the potential to overcome the challenges associated with traditional metallodrug administration, leading to more effective treatments and better patient outcomes.

Nanocarriers, such as liposomes, micelles, and polymeric nanoparticles, can enhance the solubility, stability, and bioavailability of metallodrugs by encapsulating them in hydrophilic or hydrophobic compartments, depending on their physicochemical properties (Yadav et al., 2021). This encapsulation can protect the drugs from degradation, increase their circulation time, and facilitate their accumulation in tumors through the effect of improved permeability and retention (EPR) (Maeda et al., 2013). By improving these factors, nanocarriers have the potential to increase the overall therapeutic potential of metallodrugs, resulting in more effective treatments for NSCLC patients.

Nanocarriers can be functionalized with targeting ligands, such as antibodies, peptides, or small molecules, to specifically recognize and bind to overexpressed receptors on tumor cells, thereby improving tumor targeting and reducing systemic toxicity. For example, folic acid-conjugated gold nanoparticles loaded with a gold (III) complex have demonstrated increased tumor uptake and cytotoxicity *in vitro* and *in vivo* compared to the free gold (III) complex (Yücel et al., 2020). Targeted nanocarriers can lead to the more precise delivery of metallodrugs, minimizing damage to healthy tissue and improving overall therapeutic outcomes.

Drug delivery systems have been shown to significantly improve the therapeutic potential of metallodrugs by enhancing their pharmacokinetic properties, tumor targeting, and intracellular delivery while minimizing their toxicity. These advancements are expected to facilitate the translation of novel metallodrugs into clinical practice and improve their therapeutic outcomes in NSCLC patients. As drug delivery systems continue to evolve, they are anticipated to play an increasingly important role in optimizing the use of metallodrugs to treat various cancers, including NSCLC.

Stimuli-responsive materials have been employed in drug delivery systems to enable the controlled and targeted release of metallodrugs in response to specific physiological or pathological stimuli, such as pH, temperature, enzymes, or redox conditions. For example, pH-responsive polymeric nanoparticles have been developed for the targeted delivery of cisplatin, demonstrating enhanced tumor penetration and controlled drug release in response to the acidic tumor microenvironment (Jia et al., 2022). This approach can further improve the therapeutic index of metallodrugs by ensuring selective release at the tumor site and minimizing off-target effects. Additionally, stimuli-responsive drug delivery systems can help overcome various biological barriers, such as drug resistance and limited penetration, further enhancing the therapeutic potential of metallodrugs.

Despite the significant progress made in developing advanced drug delivery systems for metallodrugs, several challenges still need to be addressed. These include optimizing the physicochemical properties of nanocarriers, controlling drug release kinetics, and ensuring biocompatibility and safety. Moreover, the translation of these novel drug delivery systems from preclinical research to clinical applications requires extensive *in vivo* studies, regulatory approval, and thorough evaluation of their therapeutic efficacy, safety, and cost-effectiveness. Nevertheless, the continued development and optimization of drug delivery systems hold great promise for enhancing the therapeutic potential of metallodrugs in the treatment of NSCLC and other malignancies.

## Discussion

### Future perspectives and challenges

Metallodrugs are ushering in a new era for NSCLC treatment. Distinct from traditional therapeutics, these metal-based agents harness unique properties like redox potential and coordination chemistry, enabling targeted interventions in NSCLC-specific molecular pathways. Unlike natural compounds, the specificity of metallodrugs is paramount due to their inherent metallic characteristics and potential side effects. As precision oncology evolves, pinpointing biomarkers for metallodrug responsiveness becomes crucial, emphasizing their distinctiveness from other drug classes.

Artificial intelligence (AI) and machine learning (ML) are revolutionizing metallodrug research. They identify patterns linking metallodrug properties to therapeutic outcomes, guiding the design of these agents for optimal efficacy and safety. Collaboration across disciplines is vital, given the challenges unique to metallodrugs, such as therapy resistance stemming from their metal core: a concern not shared with natural compounds.

The pharmacological trajectory of metallodrugs, influenced by their metal core, demands tailored evaluations. Moreover, their intricate design may command higher pricing, necessitating strategies for affordability without compromising their metal-centric benefits.

In summary, metallodrugs offer a promising avenue in NSCLC treatment, distinguished by their metal-based properties. Their potential, coupled with the challenges they present, underscores the need for specialized research and application approaches, setting them distinctly apart in the therapeutic landscape.
